# Effects of oxygen fertilization on damage reduction in flooded snap bean (*Phaseolus vulgaris* L.)

**DOI:** 10.1038/s41598-022-08165-5

**Published:** 2022-03-11

**Authors:** Danyang Liu, Anna-Lisa Paul, Kelly T. Morgan, Guodong Liu

**Affiliations:** 1 Horticultural Sciences Department, University of Florida/IFAS, 1253 Fifield Hall, 2550 Hull Road, PO Box 110690, Gainesville, FL 32611 USA; 2 Soil and Water Science Department, University of Florida/IFAS, McCarty Hall, Gainesville, FL 32611 USA; 3 University of Florida Interdisciplinary Center for Biotechnology Research, Gainesville, FL 32610 USA

**Keywords:** Plant sciences, Plant physiology

## Abstract

Flooding is one of the major abiotic stresses for vegetable production in Florida. Hydroponic and pot trials were conducted with snap bean to evaluate the effects of oxygen fertilization on the biochemical and physiological status of flooded snap bean plants. There were three treatments in the hydroponic trials were: (1) flooded (control), (2) bubble aeration with ambient air, and (3) hydrogen peroxide (H_2_O_2_) applied at the beginning of the trial. Plant health was evaluated by determining nitrogen (N) and phosphorus (P) uptake rates. The greenhouse pot trials were used to quantify the effects of three different application rates of solid oxygen fertilizers as calcium peroxide (CaO_2_) and magnesium peroxide (MgO_2_). The results showed that plant N and P uptake rates were significantly greater (p < 0.05) with H_2_O_2_ than without H_2_O_2_. The N uptake rates with H_2_O_2_ were like that of those with bubbling. The uptake rate of NH_4_^+^ was significantly greater than that of NO_3_^−^ with the bubbling and H_2_O_2_ conditions_,_ but the uptake rate of NO_3_^−^ was significantly greater than that of NH_4_^+^ in the flooding condition. The plant height, leaf greenness, shoot biomass, and yield were all significantly greater with CaO_2_ or MgO_2_ than without either solid oxygen fertilizer. The minimum damage of flooded snap bean was found with 2 g CaO_2_ or 4 g MgO_2_ per pot. These results indicated that oxygen fertilization may potentially improve yield of flooded snap bean plants.

## Introduction

Florida ranks first in acreage and total value of snap beans (*Phaseolus vulgaris*) production, but snap bean productivity is often greatly reduced by heavy rain events resulting from hurricanes and tropical storms. Snap bean is susceptible to flooding and suffers from hypoxic stresses, which results in growth suppression and yield reduction^1^. According to Holbrook and Zwieniecki^2^, the primary cause of hypoxic stress is oxygen deficiency in the soil; the oxygen diffusion coefficient is 10,000 times greater in the air (2.14 × 10^–1^ cm^2^ s^−1^) than in water (1.97 × 10^–5^ cm^2^ s^−1^). For most plant species^3^, oxygen deficiency occurs in the root zone soil with dissolved O_2_ concentrations less than 2 mg L^−1^. Thus, snap bean plants in flooded fields receive insufficient bioavailable O_2_ for normal root metabolism and suffer from waterlogging damage.

In hypoxia, anaerobic metabolism is activated, and oxidative phosphorylation is stopped. The ATP biosynthetic rate derived from glycolysis is low^4–7^. In the Krebs cycle, due to the lack of bioavailable O_2_ as the final electron acceptor, the intermediates build-up, NAD(P)^+^ levels decrease, pyruvate accumulates, and ATP concentrations decline. These changes negatively affect plant metabolism, including uptake of nutrients such as nitrogen (N) and phosphorus (P) and their assimilation^3,8^. In addition, plants suffer from ethanol accumulation and toxins formed in anaerobic metabolism^9^.

Alcohol dehydrogenase (ADH) is a well-studied enzyme in plants and is inducible in roots upon exposure to hypoxic or anaerobic conditions. When the oxygen bioavailability in the root zone is low, ADH activity increases significantly, improving the plant’s tolerance to hypoxia or anoxia^10,11^. ADH activity is considered essential for the survival of plants in hypoxic or anaerobic conditions^12^. Thus, ADH activity in root tips of flooded plants is used as an indicator of potential tolerance to flood and flooding severity.

Oxygen fertilization of the root zone is a potential method to mitigate the damage from flooding stress. There are two types of oxygen fertilizers: solid oxygen fertilizers (SOF), such as CaO_2_ and MgO_2_, and liquid oxygen fertilizers, such as hydrogen peroxide (H_2_O_2_)^13,14^. The solid oxygen fertilizers listed here release bioavailable oxygen slowly, i.e., slow-release oxygen fertilizer, whereas the liquid oxygen fertilizer, H_2_O_2_ liberates bioavailable oxygen rapidly and is considered as a fast oxygen fertilizer. Hydrogen peroxide can be decomposed biologically by the enzyme catalase (EC 1.11.1.16). The decomposition of H_2_O_2_ releases 0.5 mol of bioavailable O_2_ per mole H_2_O_2_ as shown in the equation^15^:

$${\text{H}}_{2} {\text{O}}_{2} \mathop{\longrightarrow}\limits^{Catalase}0.5{\text{O}}_{2} + {\text{H}}_{2} {\text{O}}.$$

In flooded soil, solid oxygen compounds (e.g., CaO_2_, MgO_2_) break down to H_2_O_2,_ which then provides bioavailable oxygen to the rhizosphere, as shown in the equation^1,13^:

$$\begin{gathered} {\text{CaO}}_{{2}} + {\text{ 2H}}_{{2}} {\text{O }} \to {\text{ Ca}}\left( {{\text{OH}}} \right)_{{2}} + {\text{ 2H}}_{{2}} {\text{O}}_{{2}} \hfill \\ {\text{MgO}}_{{2}} + {\text{ 2H}}_{{2}} {\text{O }} \to {\text{ Mg}}\left( {{\text{OH}}} \right)_{{2}} + {\text{ 2H}}_{{2}} {\text{O}}_{{2}} \hfill \\ \end{gathered}$$

Thus, soil amended with insoluble solid oxygen fertilizers may potentially reduce hypoxic stress in the root zone caused by flooding.

The objectives of this study were to (1) quantify the oxygen bioavailability dynamics of hydroponic solution with H_2_O_2_ application, (2) determine the effects of oxygen fertilizer application on N and P uptake by flooded snap bean plants, and (3) evaluate the effect of slow-release solid oxygen fertilizers at different rates on growth and yield of flooded snap bean.

## Results

### Effects of H_2_O_2_ application on NH_4_^+^, NO_3_, and P uptake by flooded snap beans in hydroponic solution

**Figure 1:**
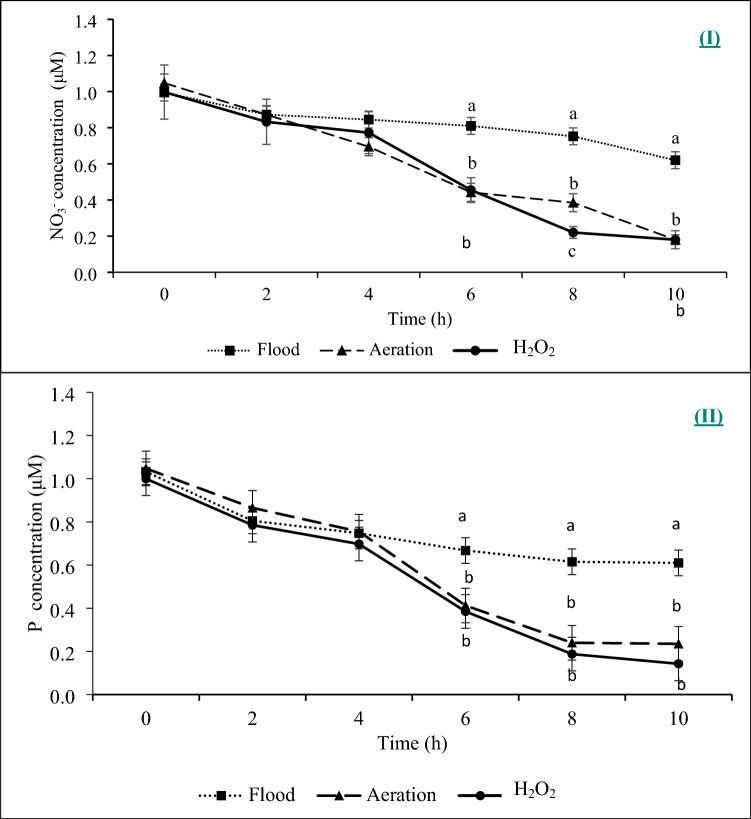
The dynamic changes of NO_3_^−^ (I) and P (II) concentrations in the measuring solution with three treatments over time: (1) flood without bubbling or H_2_O_2_ application after pre-set time for the measurement (approximately 100 μM DO level); (2) aeration (bubbling, 250 μM DO); and (3) application of 529 μM H_2_O_2_. Different letters at the same time-point indicate significant differences based on the Tukey’s Honest Significant Difference (HSD) test (p ≤ 0.05).

**Figure 2:**
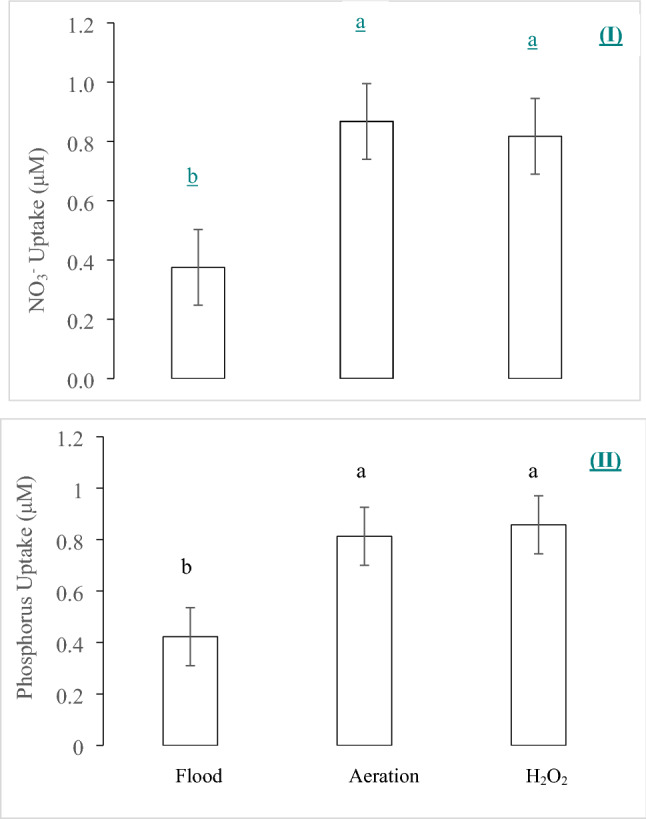
The cumulative uptake of NO_3_^−^ (I) and P (II) with three treatments for 10 h: (1) flood (approximately 100 μM DO level at 0 h); (2) aeration (bubbling, 250 μM DO); and (3) application of 529 μM H_2_O_2_. The bars with different letters are significantly different based on the Tukey’s Honest Significant Difference (HSD) test (p ≤ 0.05).

**Figure 3:**
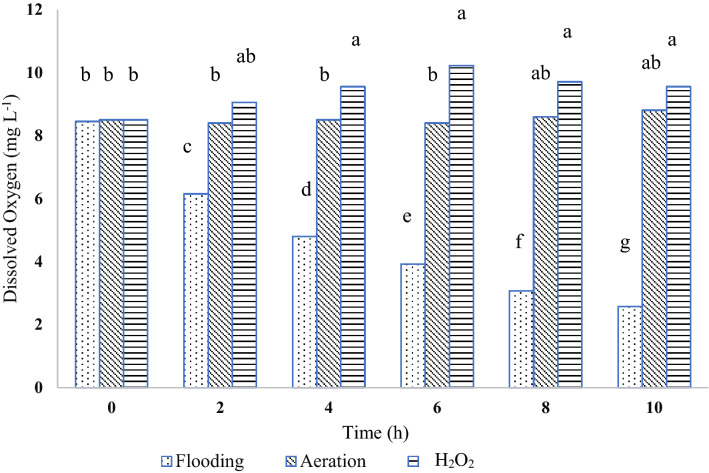
The dissolved oxygen (DO) concentrations with three treatments for 10 h: (1) flood without aeration or H_2_O_2_ application); (2) aeration (bubbling, 250 μM DO); and (3) application of 529 μM H_2_O_2_. The bars with different letters are significantly different based on the Tukey’s Honest Significant Difference (HSD) test (p ≤ 0.05).

In the hydroponic trial 1–1, “flooding” is defined as plants grown in a solution that receives neither aeration nor application of H_2_O_2_. In all the treatments, the concentrations of NO_3_^−^ and P in the nutrient solution declined over time (Fig. 1), which indicated NO_3_^−^ and P were taken up by the plant. There were differences in the nutrient uptake rates between the control and either aeration or H_2_O_2_ application. However, there was not any significant difference in the uptake rate of NO_3_^−^ between the treatments of H_2_O_2_ and aeration (Fig. 2). The NO_3_ uptake rate was approximately 50% lower with flooding than with the aeration or H_2_O_2_ treatment. Phosphorus showed a similar trend as NO_3_^−^; there was no significant difference between the H_2_O_2_ and aeration treatments, but the flooding control had significantly lower P uptake rate (Fig. 2) than each of the H_2_O_2_ and aeration treatments. The concentration of dissolved oxygen (DO) was not significantly different between the H_2_O_2_ and aeration treatments in the 10 h experiment (Fig. 3). The DO level of the control was significantly lower than each of the H_2_O_2_ and aeration treatments after 2 h. This result also indicated that the H_2_O_2_ application had a slightly better effect than aeration in oxygenating the hydroponic solution.

**Figure 4:**
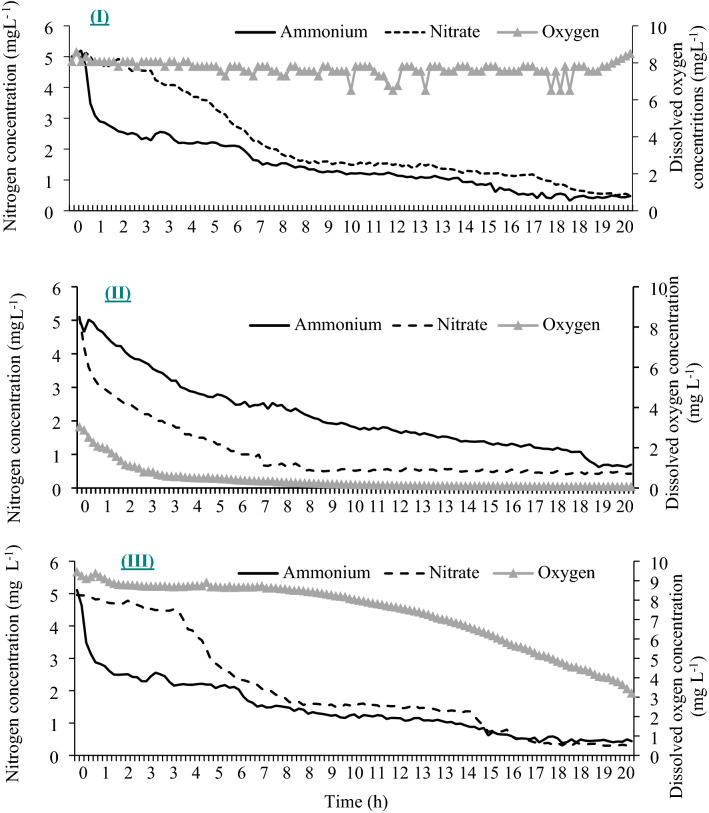
The dynamic changes for NO_3_^−^, NH_4_^+^, and DO concentrations in containers with three conditions. I = aeration condition, II = flooding conditions (starting at 3 mg L^−1^ DO level, without any oxygen supplement) for 20 h, and III = flooding conditions with 529 μM H_2_O_2_ at 0 h. *Ammonium* = NH_4_^+^ concentration, *Nitrate* = NO_3_^−^ concentration, *Oxygen* = DO concentration. The concentration readings were taken every 10 min.

In trial 1–2, the plant took up NH_4_^+^ first in the H_2_O_2_ and aeration treatments, whereas it took up NO_3_^−^ first in the flooding treatment. For the flooding treatment, the DO levels declined from 3 mg L^−1^ to approximately 0.01 mg L^−1^ from 0 to 10 h, the NO_3_^−^ and NH_4_^+^ concentrations reduced with time (Fig. 4). The depletion of NO_3_^−^ was faster than that of NH_4_^+^ from 0 to 8 h, and the NH_4_^+^ concentration remained greater than that of NO_3_^−^_._ After 10 h, the NO_3_^−^ and NH_4_^+^ concentrations were not significantly different. However, in the H_2_O_2_ and aeration treatments (Fig. 4), the concentration of NH_4_^+^ decreased faster than that of NO_3_^−^ during 0 to 8 h, and the concentration of NO_3_^−^ remained greater than that of NH_4_^+^. The DO levels of the H_2_O_2_ treatment remained greater than 8 mg L^−1^ during 0 to 10 h and declined to 3 mg L^−1^ at 20 h, which indicated that H_2_O_2_ had to be applied every 20 h to maintain a satisfactory DO concentration.

**Figure 5:**
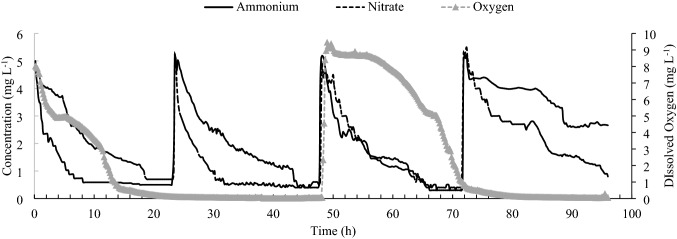
The dynamic changes of NO_3_^−^, NH_4_^+^, and DO concentrations in the container for 96 h. The solution in the container was O_2_ aerated at 0 h, and the 529 μM H_2_O_2_ was applied at 48 h. The 5 mg L^−1^ of NO_3_^−^ and NH_4_^+^ were applied every 24 h. *Ammonium* = NH_4_^+^ concentrations, *Nitrate* = NO_3_^−^ concentrations, *Oxygen* = DO concentrations. The concentration readings were taken once every 10 min.

For trial 1–3, the DO level in the solution was 8 mg L^−1^ at the beginning_,_ which was air saturated. The DO levels were consistently and significantly greater with H_2_O_2_ (Fig. 5) than with aeration (8 mg L^−1^) during 0 to 20 h. However, the DO level dropped to approximately 0.01 mg L^−1^ after 20 h, which indicated a hypoxic stress occurred before the beginning of day 2. At the beginning of day 2 before H_2_O_2_ was applied, the uptake rate of NO_3_^−^ was significantly greater than that of NH_4_^+^ which was also observed in the flooding treatment in trial 1–2 (Fig. 4). Nitrate contains 3 atoms of oxygen and can provide oxygen served as the terminal electron acceptor in cellular respiration, but ammonium does not contain any oxygen. The reversal of this trend occurred 1 h after the H_2_O_2_ was applied at the beginning of day 2 and occurred 3 h after the H_2_O_2_ was applied on day 3.

### Effects of SOF application on flooded plants in containers

For trial 2, the seedlings with SOF application had significantly more units of soil plant analysis development (SPAD) readings than those without SOF (Table 1) 2 days after flooding. The SPAD reading is an indicator of leaf greenness related to chlorophyll content in leaves. Across the different CaO_2_ rates, the SPAD readings were lower with 1 g CaO_2_ than with higher CaO_2_ rates. Application rates of MgO_2_ were not significant in leaf greenness. Furthermore, the differences in the SPAD readings of the flooded plants were also significant greater with each of 2 g, 4 g CaO_2,_ and 8 g MgO_2_ than without applying peroxide. However, the plants without flooding had significantly greater SPAD readings with each of 2 g, 4 g CaO_2,_ and 4 g, 8 g MgO_2_ than with either of 1 g CaO_2_ or no peroxide. The readings with 1 g CaO_2_ were significantly greater than the control without oxygen fertilization. No significant difference was found among 2 g and 4 g CaO_2_ and all rates of MgO_2_ (Table 1).

**Table 1:** Effects of applications of CaO_2_ and MgO_2_ on the SPAD (chlorophyll content), height, and shoot biomass of snap bean with and without flooding in pots, and data were collected two days after flooding, since at that time the flooded plants started wilting.

Treatment	Chlorophyll content (SPAD reading)		Plant height (cm)		Shoot biomass (g/plant)	
	Flooding	Non-flooding	Flooding	Non-flooding	Flooding	Non-flooding
Control	20.55c	39.56 b	17.25b	21.75 c	4.44b	7.75 b
1 g CaO_2_	28.98b	39.80 b	21.50a	24 bc	5.13ab	8.18 ab
2gCaO_2_	33.63a	42.25 ab	22.00a	25 b	5.69a	8.69 a
4gCaO_2_	34.00a	42.08 ab	21.75a	25.75 a	5.63a	8.57 ab
2gMgO_2_	34.53a	39.45 b	21.00a	24.25 bc	5.31a	8.00 ab
4gMgO_2_	37.38a	43.50 ab	21.20a	26.25 a	5.35a	8.69 a
8gMgO_2_	36.08a	44.58 a	21.00a	22.25 c	5.50a	8.25 ab
HSD_0.05_	3.6	1.41	1.55	0.84	0.67	0.27

Any two means within a column not followed by the different letter are significantly different using Tukey`s honest significant difference (HSD) test at p < 0.05.

The flooded plants were significantly taller with CaO_2_ or MgO_2_ than without peroxide application. Plant height without flooding was significantly taller with oxygen fertilizer than without oxygen fertilizer. The non-flooded plants with 4 g CaO_2_ and 4 g MgO_2_ had the highest height (Table 1).

Shoot biomass of flooded plants was greater with CaO_2_ or MgO_2_ than without SOF (Table 1). In the non-flooding treatment, only those with 2 g MgO_2_ and without SOF had the lowest plant biomass (Table 1).

**Figure 6:**
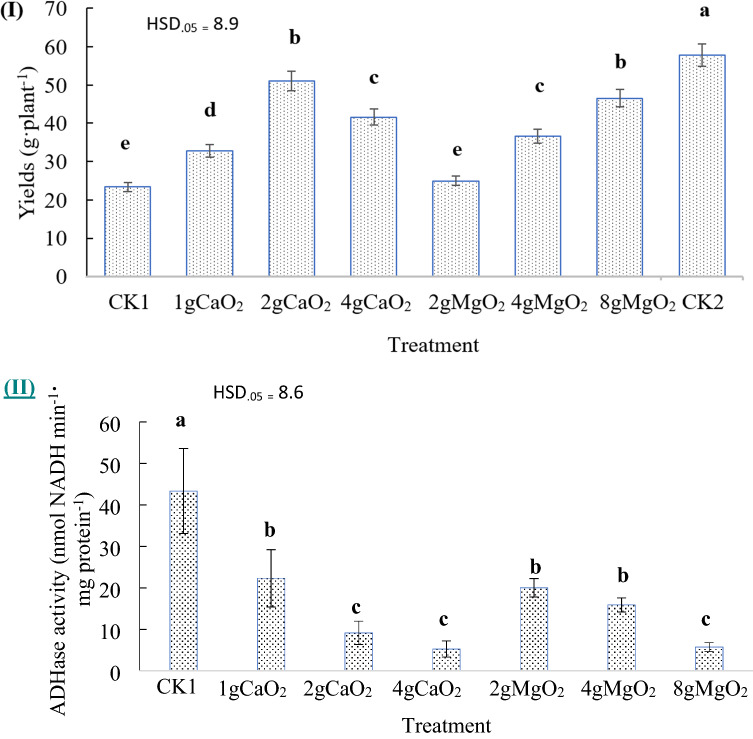
Effects of flooding and applications of CaO_2_ and MgO_2_ on the yields (I) and ADH (II) of snap bean in pots. The horizontal axis shows different amount of CaO_2_ and MgO_2_ applied. CK1 represents the group without CaO_2_ and MgO_2_ with flooding, CK2 represents the group without CaO_2_ and MgO_2_ and without flooding. The bins with different letters are significantly different at p ≤ 0.05.

The pod yields of flooded snap bean were greater with SOF treatments than the control (Fig. 6). The 2 g CaO_2_ and 8 g MgO_2_ treatments had almost 50% greater yield than the control, which was significantly greater than that of rest of the treatments (Fig. 6).

As a bio-indicator, ADH activity is used to evaluate the effects of different amounts of SOF on the growth and development of flooded plants. The ADH activities were significantly lower with all the SOF treatments than with the flooding treatment. For example, ADH activity was in a range from 44 nmol NADH per minute per mg protein for the flooded snap bean plants without oxygen fertilization to 5 nmol NADH per minute per mg protein for those flooded plants with 4 g CaO_2_ (Fig. 6). The lower ADH activities indicated the SOF treatments had much less flooding stress compared to the flooding treatment. the treatments of 2 g CaO_2_, 4 g CaO_2_, and 8 g MgO_2_ had significantly lower ADH activities than the other treatments (Fig. 6), which indicated those treatments had even less or no flooding stress compared to the other treatments. This result provided physiologic evidence for evaluation of the potential of SOF on mitigating the flooding stress.

## Discussion

In trial 1, the H_2_O_2_ treatment had a positive effect on the N and P uptake of the plants in hypoxic stress. The lower uptake rate of flooded plants was possibly caused by ATP shortage, resulting from flooding-induced oxygen deficiency and anaerobic respiration. Thus, the application of H_2_O_2_, supplying O_2_ directly, was a potential method to alleviate the hypoxia damage. This finding is supported by other studies. Jampeetong and Brix^16^ found that *Salvinia natans* plants supplied with NO_3_^−^ grew normally in a hypoxic solution but were greatly inhibited when only NH_4_^+^ was supplied as the nitrogen source.

In trial 2, the results showed that the application of SOFs enhanced the growth and development of snap bean plants in flooding stress. Lakitan^17^ reported that the total yield and survival of snap beans were susceptible to flooding imposed after reproductive development had begun (28 DAP), and flooding imposed at later growth (> 28 DAP) significantly reduced the total yields. Few plants survived, and there was no measurable yield when flooding was imposed at 36 DAP. More trials using SOF with different flood durations need to be conducted at various plants growth stages in the future.

After uptake, nitrogen assimilation follows a few steps including from the reduction of NO_3_^−^ to NH_4_^+^ and the subsequent incorporation of NH_4_^+^ into amino acids^18^. NO_3_^−^ reduction is catalyzed by the enzyme nitrate reductase (NR, EC 1.6.6.1-3). Nitrate reductase reduces NO_3_^−^ to NO_2_^−^, which is then transferred to the chloroplast to be reduced to NH_4_^+^ by nitrite reductase (NiR, EC 1.6.6.4). After that, NH_4_^+^ is used to biosynthesize glutamine by glutamine synthase (GS, EC 6.3.5.1)^19^. Nitrate reductase is a key enzyme tightly regulated by many environmental factors, such as flooding^20,21^. Anoxia and hypoxia increase NR activity, and NO_3_^−^ without available molecular oxygen is used to oxidize organic molecules to obtain energy, a phenomenon called “nitrate respiration”^22^. That study may support the result that snap bean absorbs more NO_3_^−^ than NH_4_^+^ in hypoxia because the increased NR and NiR activity would promote NO_3_^−^ uptake and assimilation. Early trials used labeled^15^ NO_3_^−^ to monitor nitrate assimilation during anaerobic germination of rice and showed that ^15^N was incorporated into amino acids^23^. The results confirmed that exogenous NO_3_^−^ supplied during oxygen deficiency had been assimilated by plants^23^. Even if NO_3_^−^ supply to flooded crops increases the percentage of survival, the biochemical and molecular mechanisms underneath its positive effects are not fully understood. Thus, future research will be needed to invest the mechanisms for plant surviving from flooding with NO_3_^−^ amendment.

The results of this study showed the trend that the supplement of NO_3_^−^ may enhance the NH_4_^+^ uptake (Fig. 4). For example, ample supply of NO_3_^−^ was able to alleviate the consequences of root anoxia or hypoxia in barley^9^. It has been hypothesized that NO_3_^−^ can serve as an alternative terminal electron acceptor in hypoxic or anoxic conditions^8^. Nitrate may alleviate the consequences of anoxia in other ways. As an ample supply of NO_3_^−^ can directly induce nitrate reductase activity^24^, whereby NADH is diverted from the reduction of acetaldehyde to ethanol. The accumulation of injurious ethanol concentrations in the cells is delayed. Also, NO_3_^−^ may influence rhizosphere redox potential and may act as an oxygen source through influx into O_2_-deficient roots or by uptake in partially aerated shallow roots^9^.

The results of this study indicated that the peroxides, CaO_2_ and MgO_2_ have the potential to mitigate the flooding damage in snap beans. Several researchers have observed that prolonged flooding causes a cessation of root and shoot growth, wilting, decreased nutrient uptake, and often plant death^25^. According to Lakitan^17^ flooding on different growth stages of snap bean had different effects on growth and yield, and there is evidence that longer flooding duration would result in lower survival and yields^17^. Thus, further studies are warranted to assess the effects of solid oxygen fertilizations on reducing flooding stress, and survival rates of snap beans at different growth stages and with different duration of flooding treatments.

## Conclusions

Snap bean is susceptible to hypoxic stress and often suffers from flooding, particularly in the hurricane and tropical storm seasons. We conducted both hydroponic and pot trials to investigate how oxygen fertilization affects uptake rates of NH_4_^−^ and NO_3_^−^ and P, growth, and yields of flooded snap bean. Three peroxides, including H_2_O_2_, CaO_2_, and MgO_2_, were evaluated. The findings from this study indicated oxygen fertilization had the potential to minimize flooding damage to flooded snap bean. Hydrogen peroxide application significantly increased the dissolved oxygen level and the uptake rate of NH_4_^+^, NO_3_^−^ and P by snap bean. Snap bean had an uptake preference for of NH_4_^+^-N with sufficient bioavailable oxygen. However, NO_3_-N is preferred in flooding conditions. The right dosage of solid oxygen fertilizer of 2 g CaO_2_ or 8 g MgO_2_ had the greatest yield among all flooded snap bean plants. Thus, both CaO_2_ and MgO_2_ oxygen fertilizations had the potential to keep absorbing nutrients such as N and P by flooded snap bean plants, reduce flooding damage, and minimize the yield loss. These novel methods of oxygen fertilization may mitigate economic loss and benefit agriculture often facing different hypoxic stresses.

## Materials and methods

### Plant materials and soil

Snap bean (P. vulgaris. cv. ‘Bronco’) is a primary vegetable crop and widely cultivated in USA. The ‘Bronco’ seeds are commercially available for research and breeding and were purchased from a seed company, HOSS TOOLS located in Norman Park, GA (GPS coordinates: Latitude: 31.2462 and Longitude -83.6549) before the study was started in spring 2015. Miracle-Gro® garden soil was procured from Lowes in Gainesville, FL.

In Trial 1, snap bean seeds were soaked in 0.15% H_2_O_2_ for 24 h and rinsed with deionized (DI) water for 5 times, and then were placed into a 12-cm petri dish with moistened filter paper and sealed inside a zip-lock plastic bag and incubated at 33 °C overnight. After seedling emergence, each seedling was transplanted into a 5.1-cm diameter plastic mesh basket. The baskets (Net Pots Net Cups, Heavy Duty Plastic Net Pot with Wide Rim Design) were placed in an aeroponic system to allow for rapid root growth and development. The aeroponic system consisted of a tank half-filled with 10% Hoagland solution^26^, a misting pump, tubing, and three sprinklers that continuously misted the roots. After two weeks, the seedlings with three true leaves were transferred from the aeroponic system to a hydroponic system where the roots were submerged in a 1000 mL 0.2 mM CaSO_4_ solution for measurement use. The use of 0.2 mM CaSO_4_ solution was to protect the membranes, since calcium is essential for the intactness and selectivity of biological membranes^27^.

In trial 2, two sizes of pots were used for different purposes. The smaller pots with 7.6 cm in diameter × 8.9 cm in height was for seed germination. The larger pots with 15 cm in diameter × 16.5 cm in height for production. Miracle-Gro® garden soil was used, and the fertilizers were applied per pot as follows: NH_4_NO_3_, 2.92 g; triple superphosphate, 1.65 g; potassium chloride, 0.44 g per pot.

#### Experimental design

The trials were all conducted in the Horticultural Sciences Department, University of Florida/IFAS, Gainesville, Florida. The hydroponic trials had three sub-trials. Trial 1–1 and 1–2 had the same treatments with four replicates. The trial 1–1 was conducted in an environmental-controlled growth chamber with a temperature range of 23–27 °C, 16-h daylight and 8-h dark. The trial 1–2 and trial 1–3 were conducted in a standard greenhouse with a temperature range of 23–27 °C. The trial 2 was conducted in a high tunnel under a natural atmosphere. The treatments were imposed by (1) flooding control without bubbling or applying H_2_O_2_ after the pre-set time (approximately 100 μM DO level at the beginning, no oxygen supplement), (2) aeration (continuous oxygen supply with an air pump), and (3) H_2_O_2_ (application of 529 μM H_2_O_2_ at 0 h). The growth medium was 1 L DI water with 0.22 g L^−1^ KH_2_PO_4_, 0.373 g L^−1^ KNO_3_, 0.02 g L^−1^ CaSO_4,_ 0.012 gL^−1^ MgSO_4_, 0.29 mg L^−1^ H_3_BO_3_, 0.18 mg L^−1^ MnCl_2_∙4H_2_O, 0.02 mg L^−1^ ZnSO_4_∙7H_2_O, 0.008 mg L^−1^ CuSO_4_∙5H_2_O, 0.002 mg L^−1^ H_2_MoO_4_∙H_2_O, 0.65 mg L^−1^ Ethylenediaminetetraacetic acid (EDTA), and 0.62 mg L^−1^ FeSO_4_∙7H_2_O. For trial 1–1, 2 mL samples were collected from the center of pots every 2 h for 10 h. The samples were analyzed for ortho-P (EPA Method 365.3), and NO_3_^−^ (EPA Method 352.1) using a Seal AQ-2 discrete analyzer (2006 SEAL Analytical Ltd. Mequon, WI) (Fig. 1).

**Figure 7:**
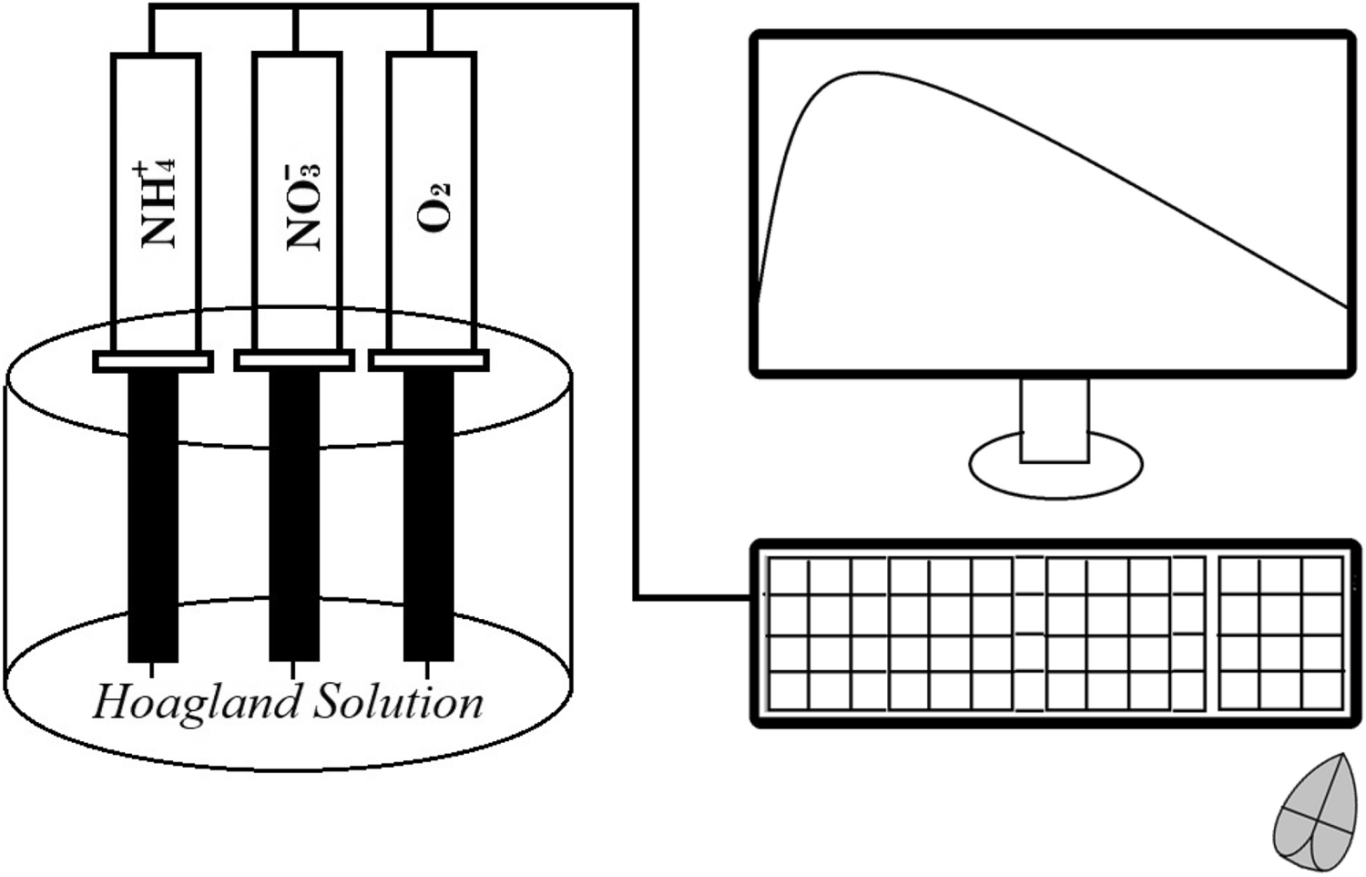
The diagram of container and sensors used in monitoring changes in concentrations of NH_4_^+^, NO_3_^−^, and DO in hydroponics with a snap bean plant.

For trials 1–2, Neulog™ Dissolved oxygen sensors, Neulog NO_3_^−^ sensors, and Neulog NH_4_^+^ sensors were set up for each container to monitor concentration changes (Fig. 7). Those three sensors were combined and installed in each container, and aluminum foil was used to hold the sensors and plants and block the sunlight. The data were monitored every 10 min for 20 h and recorded with NeuLog™ Dissolved Oxygen sensor, Nitrate logger sensors, and Ammonium logger sensors (Neulog brand dissolved oxygen sensor, EISCO Scientific, Rochester, NY, USA).

Trials 1–3 used the same sensors and settings as trials 1–2 to monitor the consistently dynamic nitrogen changes for 96 h (96 h). The growth medium was 1 L DI water with the same ingredients as trial 1–1 but used 0.57 g L^−1^ NH_4_H_2_PO_4_ and 1.8 g L^−1^ KNO_3_ instead of 0.22 g L^−1^ KH_2_PO_4_ and 0.373 g L^−1^ KNO_3_. Additionally, 0.57 g L^−1^ NH_4_H_2_PO_4_ and 1.8 g L^−1^ KNO_3_ were applied every 24 h to provide 5 mg L^−1^ of NO_3_^−^ and 5 mg L^−1^ of NH_4_^+^. The data were recorded every 30 min for 4 days (96 h).

In trial 2, a completely randomized design was used with 4 replications. There were two SOFs at different rates, i.e., CaO_2_ at 0, 1, 2, and 4 g per pot and MgO_2_ at 0, 2, 4, and 8 g per pot. The seeds were planted in 7.6 cm pots for two weeks and were transferred to the 15.2-cm pots (internal volume: 2,622 cm^3^) after germination. The SOF, CaO_2_ or MgO_2_ was mixed with the soil (approximate 2,000 cm^3^ per pot), the mixed soil was added into the 15.2-cm pots, and then the seedlings were transplanted into the pots, respectively. One seedling was planted per pot. The fertilizer rates were based on the vegetable production handbook of Florida^28^. The N, P and K application rates were 112 kg ha^−1^, 134 kg ha^−1^ and 134 kg ha^−1^ for the whole season. After one week, both groups with CaO_2_ and MgO_2_ were each divided into two subgroups. One subgroup was individually flooded in 18.9 L buckets filled with water to simulate the flooding conditions for two days, while another subgroup was not flooded.

The data of leaf greenness representing leaf chlorophyll content determined by a SPAD meter (Knoica-Minolta, Osaka, Japan), plant height, ADH activities, and shoot biomass were measured when the flooded plants started wilting. To collect yield data, this trial was identically repeated and the yields were measured 55 DAP.

#### Root ADH activity

In trial 2, two days after flooding, approximately 1 g of roots (4–5 cm from the tips) was harvested per treatment and placed in a container filled with liquid nitrogen. Root ADH activity was determined according to a modification of the method^30^ as follows. The root tips (1 g) were homogenized in an extraction solution buffer (10 ml) composed of 7.88 g L^−1^ Tris–HCl (pH 8.0), 0.29 g L^−1^ EDTA, 0.5 μg L^−1^ DTT, and 0.08 mg L^−1^ β-mercaptoethanol. Then, the extraction solution of the ADH enzyme was centrifuged at 4 °C at 15,000 rpm for 10 min. 100 μL of enzyme solution was added to 900 μL of a reaction solution composed of 7.88 g L^−1^ Tris–HCl (pH 9.0), 0.29 g L^−1^ EDTA, and 0.66 g L^−1^ NAD. The mixture was incubated in 1.5–mL microcentrifuge tubes in a water bath at 30 °C for 3 min. To start the measurable reaction, 100 µL of 100% ethanol was added to the 900 µL reaction mixture. Measurements were taken after 15 s, and the absorbance at A340 was recorded every 15 s for two minutes. After a reaction time of 1 min in the cuvette, reading at 340 nm was made with a spectrophotometer (Eppendorf BioSpectrometer®, Hauppauge, NY.USA) to determine the concentration of NADH. The activity was calculated using a value of 6.22 mmol^−1^ cm^−1^ as the molar extinction coefficient of NADH at 340 nm^31^.

#### Statistical analysis

All statistical analyses were performed using R Studio statistical software (RSTUDIO, INC., Boston, MA). One-way ANOVA was applied to test the significance of SPAD readings, plant heights, shoot biomass, yield, and ADH activities. Comparisons of means for significant effects were determined using Tukey HSD tests. The data collected were all subjected to statistical analysis through ANOVA, and the value of (HSD at 5% was calculated to compare every two means. Differences among the means and correlation coefficients were deemed statistically significant at the p < 0.05 level.

### Permission statement

All the experiments on plants, including the collection of snap bean materials, ‘Bronco’ used as the cultivar, were strictly performed in accordance with relevant guidelines, regulations, and legislation.
